# Short-long term near infrared spectroscopy patterns after different milk regimens olfactory stimuli in late preterms

**DOI:** 10.1186/s13052-025-01912-0

**Published:** 2025-04-12

**Authors:** Caterina Di Battista, Alice Grometto, Mariachiara Strozzi, Ebe D’Adamo, Giuseppe Lapergola, Antonio Maconi, Claudia Pelazzo, Marta Pasino, Vincenzo Salvo, Francesca Gazzolo, Martina Spinelli, Marta Betti, Marinella Bertolotti, Ali Saber Abdelhameed, Simonetta Picone, Diego Gazzolo

**Affiliations:** 1 Neonatal Intensive Care Unit, G. d’Annunzio University, Chieti, I-65100 Italy; 2 Neonatal Intensive Care Unit, ASO SS Antonio, Biagio, Alessandria, C. Arrigo Italy; 3 Department of Pediatrics and Neonatology, Ospedale Cardinal Massaia, Asti, Italy; 4 Integrated Activities Research Innovation Department, ASO SS Antonio, Biagio, Alessandria, C. Arrigo Italy; 5 Neonatal Intensive Care Unit, Department of Human Pathology in Adult and Developmental Age “Gaetano Barresi”, University of Messina, Messina, Italy; 6 Pediatric Unit, Department of Medical and Surgical Sciences, Magna Graecia University, Catanzaro, Italy; 7 Department of Science and Technological Innovation, Piemonte Orientale University, Alessandria, Italy; 8 Neonatology and Neonatal Intensive Care Unit, Policlinico Casilino General Hospital, Rome, Italy; 9 Department of Pharmaceutical Chemistry, College of Pharmacy, King Saud University, P.O, Riyadh, Saudi Arabia

**Keywords:** NIRS, Brain, Kidney, Splanchnic, Premature

## Abstract

**Background:**

The aim of the study is to investigate the effects of olfactory stimuli from breast and formula milk on near-infrared spectroscopy (NIRS) cerebral and splanchnic patterns in late preterm infants.

**Methods:**

We conducted a multicenter prospective observational pretest-test study in 30 late preterm infants subjected to olfactory stimuli from breast and formula milk. Regional oxygenation status, tissue function in cerebral and splanchnic districts, and cerebral-splanchnic hemodynamic redistribution were recorded at four pre-determined time-points: before sniffing (30 min), during sniffing (30 s), short-term (30 min), and long-term after olfactory stimuli (180 min).

**Results:**

After olfactory stimuli from breast and formula milk we found: (i) a significant increase (*p* < 0.05) in cerebral oxygenation and cerebral-splanchnic hemodynamic redistribution after breast milk stimulus, (ii) a significant increase (*p* < 0.05) in splanchnic oxygenation and splanchnic-cerebral hemodynamic redistribution after formula milk stimulus.

**Conclusions:**

The present results show early changes in NIRS patterns in cerebral and splanchnic districts after breast and formula milk stimuli. Data opens the way to further studies using NIRS as a reliable tool for central nervous system and splanchnic development and response after olfactory stimuli.

**Supplementary Information:**

The online version contains supplementary material available at 10.1186/s13052-025-01912-0.

## Background

There is a general consensus on the unique properties of breast milk (BM) [1, 2]. BM-fed infants have a lower incidence of early and late onset sepsis, of necrotizing enterocolitis as well as better neurocognitive development [3, 4]. This especially refers to sensor cortical activation and olfaction. There is evidence that olfaction is essential for newborns: (i) behavioral adaptation, (ii) moving towards odors produced by the mother, particularly for nipple localization and feeding, and (iii) ability to identify pleasant or unpleasant smells commonly met during a stay in the neonatal intensive care unit (NICU) [5–8].

In the perinatal period, among different monitoring strategies for olfaction function, near infrared spectroscopy (NIRS) has been argued for [9, 10]. Changes in cerebral regional oxygen saturation (CrSO_2_), in fraction of tissue oxygen extraction (FTOE) and hemodynamics have been shown under different conditions [11, 12]. These mostly regarded the studied populations (preterm vs. late preterm), feeding modalities (bolus vs. continuous), feeding regimens (human vs. formula milk, FM), and the occurrence of acute/chronic hypoxia (perinatal asphyxia; intrauterine growth retardation, IUGR) [11, 13–18]. Exposure to detergent, vanilla and BM smell has been found to affect CrSO_2_, FTOE and cerebral/systemic hemodynamics [6, 19]. Recently, it has been shown that low/very low birthweight infants can process low concentrations of maternal smells at a cortical level, suggesting awareness of their environment [11, 12]. However, data on potential changes, due to BM vs. FM olfaction stimuli, in cerebral and systemic districts (i.e. splanchnic) oxygenation status, tissue function, and hemodynamic redistribution is at this stage lacking.

Therefore, in the present study we aimed to investigate, in a cohort of late preterm (LP) infants, whether BM and FM olfaction stimuli can change at short/long-term cerebral and splanchnic: (i) oxygenation status, (ii) tissue function, and (iii) systemic/splanchnic redistribution by means of NIRS longitudinal monitoring.

## Methods

### Population

We conducted an observational pretest-test study, where each case acted as its own control, in 30 LP newborns admitted to our 3rd level NICUs from June 2020 to January 2022 fed under BM and FM regimens.

The study was approved by the local ethics committees and the parents of the subjects admitted to the study gave signed and informed consent (ASO.Neonat.12.01).

Gestational age (GA) was determined by the last menstrual period and confirmed by a first trimester ultrasound scan. Appropriate growth was defined by the presence of ultrasonographic signs (biparietal diameter and abdominal circumference between the 10th and 90th centiles), according to the nomograms of Campbell and Thoms, and by post-natal confirmation of a birthweight (BW) between the 10th and 90th centiles, according to our population standards, correcting for the mother^’^s height, weight, parity, and the sex of the newborn [20, 21].

Exclusion criteria were: congenital abnormalities, perinatal asphyxia, the need for cardiovascular support, necrotizing enterocolitis, gastrointestinal anomalies and cutaneous diseases impeding the placement of probes.

### NIRS monitoring

The NIRS parameters in the cerebral (C) and splanchnic (S) districts were recorded by the Sen Smart X-100 (Nonin Medical, Minnesota, USA). Equanox Advance self-adhesive sensors (Nonin Medical, Minnesota, USA) were placed on the central region of the infants’ skulls, and on the infra-umbilical abdomen region. CrSO_2_ and splanchnic regional oxygen saturation (SrSO_2_) were calculated by the inbuilt software as well as arterial oxygen saturation (SaO_2_). Fractional tissue oxygen extraction (FTOE) represents a composite parameter and can be calculated from regional oxygen saturation (rSO2) and SaO_2_. FTOE values in the C and S districts were assessed according to the following formula: (SaO_2_ - rSO_2_)/SaO_2_ [11–18].

We also calculated the C/S oxygenation ratio (CSOR), which is the ratio of C vs. S district oximetry, according to the following formula: CrSO_2_/SrSO_2_ [22]. This ratio has been found to be a valuable index of hemodynamic redistribution in chronic hypoxic infants [23].

NIRS monitoring patterns were consecutively recorded in each LP at four different monitoring time-points: 30 min before sniffing (T0), at sniffing exposure (30 s), the short-term effects (30 min) after olfaction stimuli (T1) and, long-term effects (30 min after T1) (T2).

For each NIRS variable (C, S, rSO_2_, FTOE, CSOR), we calculated the mean ± SD from the selected 30” periods (120 data points per hour), which were chosen at the end of T0-T2. We made this choice to obtain the highest stability of the NIRS signal, even though selecting a 30” period could induce a selection bias. Sometimes this selection was not possible due to the occurrence of unwanted artifacts (generally infant movements); in this case the 30” period without artifacts closest to the end of T0-T2 was selected.

### Olfaction stimuli

To match the LP response to BM and FM olfaction stimuli, we used a cotton cloth routinely used in the NICU. Briefly, after feeding LP were positioned in a clean incubator or in an open bed in a quiet and well-ventilated single room where NIRS recordings were performed. The aim was to avoid any bias due to nosocomial odors and NICU sensory inputs. LP were tested 30 min after feeding when pre-test monitoring started (T0). The olfaction stimuli were randomly performed by putting cloths one centimeter below LP’s nose by a single examiner who was blinded to its nature. We started first with BM or FM olfaction stimuli with a presentation lasting 30 s followed by short-long term NIRS monitoring (T1, T2). Secondly, 30 min after feeding, in the same studied population, we re-started pre-test monitoring (T0) followed by FM or BM olfaction stimuli, identically performed as before, followed by T1 and T2 NIRS monitoring.

### Standard monitoring parameters

Heart and respiratory rates and SaO_2_ monitoring were continuously recorded by MX700 monitors (Philips, Eindhoven, The Netherlands) at 12” intervals. Laboratory parameters (red blood cell count, RBC; hemoglobin blood concentrations, Hb; hematocrit rate, Ht; venous blood pH; partial carbon dioxide venous pressure, pCO_2_; partial oxygen venous pressure, pO_2_; base excess, BE; blood ion, glucose, urea, creatinine and bilirubin levels) were recorded in all infants on admission to NICUs and at different NIRS monitoring time-points.

### Cranial assessment and neurological examination

Cerebral ultrasound scanning recordings were performed by a real-time ultrasound machine (Acuson 128SP5, Mountain View, CA, USA), at 12-h and 24-h from admission and on discharge from the hospital. Cerebral ultrasound scanning and neurological patterns were assessed by a single examiner in each center, blinded to the NIRS test results.

Neonatal neurological conditions were assessed daily and classified as described by Prechtl [24]. Each infant was defined as normal, suspect, or abnormal, in accordance with the classification used by Jurgens–van der Zee et al. [25].

### Perinatal outcomes

The following main perinatal and neonatal outcomes were recorded in the studied groups: maternal age; incidence of premature rupture of membrane, chorioamnionitis, gestational diabetes mellitus, preeclampsia, and IUGR; antenatal glucocorticoids supplementation; GA; BW; delivery mode; gender; Apgar scores at 1st and 5th min; occurrence of respiratory distress syndrome (RDS); need for mechanical (MV) or non-invasive (NIV) ventilation and surfactant administration; bronchopulmonary dysplasia (BPD) (moderate/severe degree) in accordance with Jobe and Bancalari [26]; persistent patent ductus arteriosus (PDA); intraventricular hemorrhage (IVH) according to Papile et al. [27]; periventricular leukomalacia (PVL); early/late onset sepsis (EOS, LOS); perinatal death; retinopathy of prematurity (ROP) (higher than second degree) [28].

### Statistical analysis

**Fig. 1:**
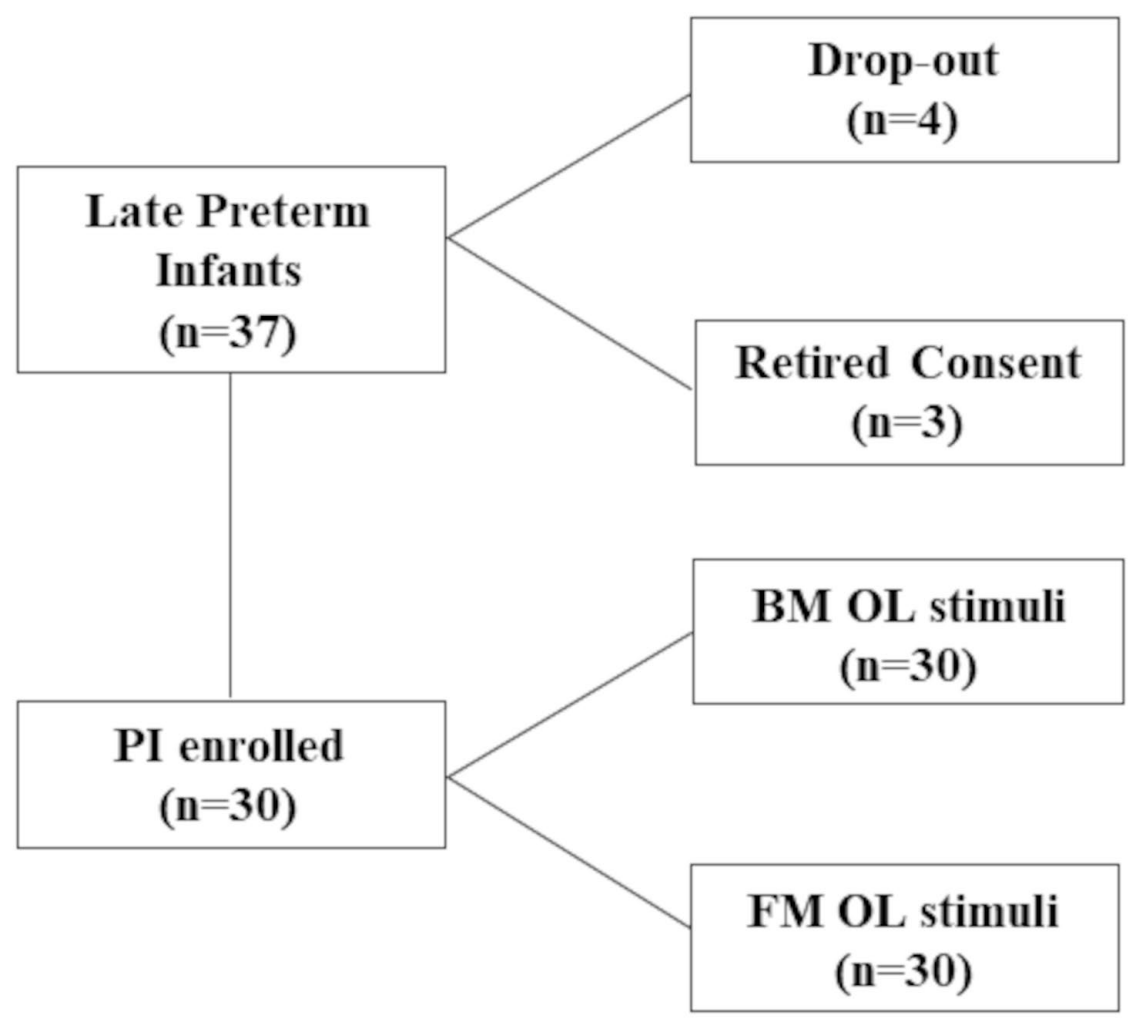
Study population. Flow chart describing patients’ recruitment

For sample size calculation, we used changes in CrSO_2_ as the main parameter [29]. We assumed an increase of 0.5 SD in CrSO_2_ to be clinically significant. Considering an α = 0.05 and using a two-sided test, we estimated a power of 0.90, recruiting 23 LP. We added *n* = 7 cases to allow for dropouts and consent retirement. Therefore, the study population was composed of 30 LP exposed to BM and FM olfaction stimuli (Fig. 1).

All perinatal and neonatal parameters were expressed in medians and interquartile ranges. Data was analyzed for statistically significant differences between the two groups, by t-test and with Mann-Whitney U two-sided test when they were not normally distributed. The comparison between the different monitoring timepoints was performed by Kruskal-Wallis one-way ANOVA. Statistical significance was established at *P* < 0.05.

## **Results**

### Population

Perinatal characteristics are reported in Table 1. Maternal age ranged from 29 to 37 years, premature rupture of membrane and chorioamnionitis incidences accounted for 26.3%, and none was complicated by gestational diabetes mellitus. Preeclampsia and IUGR rates were 10% and 3.4%, respectively. Antenatal steroid prophylaxis was administered in 23.3% of the pregnant women.

### Neonatal outcomes

GA at birth ranged from 34 to 36 weeks and all LP recruited weighed less than 2500 g. Caesarean section and vaginal delivery rates were 43.3% and 56.7% respectively, whilst male and female births accounted for 53.3% and 46.7%, respectively. Outborn/inborn incidences were 26.7% and 73.3%, respectively. None of the 30 LP showed an Apgar score < 7 at 1st and 5th minute.

Incidences of RDS and need for MV occurred in 10%, whilst rates for NIV and the need for surfactant administration 80% and 40%, respectively. None of LP were complicated by BPD, IVH, PVL, NEC, early neonatal death, and ROP. PDA incidence was 10% whilst rates of EOS and LOS were 16.7% and 6.7%, respectively. None of the LP showed overt neurological abnormalities either at pre-determined monitoring timepoints or at discharge from hospital (Table 1).

**Table 1:** Perinatal characteristics and neonatal outcomes in the late preterm infants admitted into the study

Parameters	Late preterm (*N* = 30)
*Perinatal characteristics*	
Maternal age (y)	34 ± 5
Premature rupture of membrane (n/tot)	8/30
Chorioamnionitis (n/tot)	8/30
Gestational diabetes mellitus (n/tot)	0/30
Preeclampsia (n/tot)	3/30
IUGR	1/30
Glucocorticoids (n/tot)	7/30
*Neonatal characteristics*	
GA (wks)	34 ± 1
BW (g)	2076 ± 378
CS (n/tot)	13/30
Gender (M/F)	16/14
Outborn/inborn (n/tot)	8/30
Apgar 1’	9 ± 1
Apgar 5’	9 ± 1
*Main outcome measures (n/tot)*	
RDS	3/30
MV	3/30
NIV	24/30
Surfactant administration	12/30
BPD	0/30
PDA	3/30
IVH	0/30
PVL	0/30
NEC	0/30
Death	0/30
EOS	5/30
LOS	2/30
ROP	0/30
*Neurological examination (n/tot)*	
normal/suspect/abnormal	30/0/0
normal/suspect/abnormal at discharge	30/0/0

Abbreviations: intrauterine growth retardation (IUGR); gestational age (GA); birthweight (BW); caesarean section (CS); respiratory distress syndrome (RDS); mechanical ventilation (MV); non-invasive ventilation (NIV); broncho-pulmonary dysplasia (BPD); patent ductus arteriosus (PDA); intraventricular hemorrhage (IVH); periventricular leukomalacia (PVL); necrotizing enterocolitis (NEC); early-onset sepsis (EOS); late-onset sepsis (LOS); retinopathy of prematurity (ROP); years (y); weeks (wks); grams (g)

### Monitoring parameters

Clinical (GA, W), laboratory and monitoring parameters performed at birth (heart rate, respiratory rate, SaO_2_) were within reference ranges and no significant differences (*P* > 0.05, for all) were found at T0-T2 (data not shown). Identically, blood pH, pCO_2_, pO_2_, BE, RBC, Hb, Ht, glucose, urea, creatinine, ions and bilirubin blood levels performed at pretest time-point were within reference curves (Table 2). Furthermore, no differences were found in laboratory analytes at T0-T2 (*P* > 0.05, for all) (data not shown).

**Table 2:** Monitoring parameters recorded before sniffing stimuli in late preterm infants admitted into the study

Parameter	Late Preterm (*N* = 30)
GA (wks)	36 ± 1
W (g)	2112 ± 223
pH > 7.20 (n/total)	40/40
pCO_2_ (mmHg)	41.6 ± 2.5
pO_2_ (mmHg)	43.7 ± 1.3
BE (mmol/L)	-0.9 ± 0.8
RBC count (10^12^/L)	4.2 ± 0.2
Hb (g/L)	14.1 ± 2.8
Ht (%)	40.8 ± 0.08
Plasma glucose (mmol/L)	5.3 ± 0.4
Urea (mg/dL)	36.1 ± 4
Creatinine (mg/dL)	0.79 ± 0.18
Na^+^ (mmol/L)	139 ± 1
Ca^++^ (mmol/L)	1.14 ± 0.03
K^+^ (mmol/L)	3.9 ± 0.3
Bilirubinemia (mg/dL)	6.4 ± 5.2
SaO_2_ (%)	98 ± 1

Abbreviations: gestational age (GA); weight (W); partial carbon dioxide venous pressure (pCO_2_); partial oxygen venous pressure (pO_2_); base excess (BE); red blood cell count (RBC); hemoglobin blood concentrations (Hb); hematocrit rate (Ht); pulsed arterial oxygen saturation (SaO_2_); weeks (wks); grams (g)

### NIRS monitoring after BM stimuli

In LP subjected to BM olfaction stimuli the BMCrSO_2_ pattern was characterized by a progressive increase with significantly higher (*P* < 0.05) values at T2 than T0. No differences (*P* > 0.05, for both) were found between T1 vs. T0 and T2 vs. T1, respectively. BM C FTOE at T2 was significantly lower (*P* < 0.05, for both) than T0 and T1, whilst no differences (*P* > 0.05) were observed between T0 and T1 (Table 3).

**Table 3:** Cerebral and splanchnic NIRS patterns recorded before (T0), during (T1) and after (T2) breast (BM) and formula milk (FM) olfactory stimuli in late preterm infants

	BM (*n* = 30)			FM (*n* = 30)			*P* (BM vs. FM)
Parameter	Median	25°	75°	Median	25°	75°	
CrSO_2_ T0	81	78	83	80	78	82	< 0.05
CrSO_2_ T1	80	80	83	80	78	83	NS
CrSO_2_ T2	82^**a**^	79	85	81^**a**^	78	83	< 0.05
SrSO_2_ T0	82	74	86	83	77	88	< 0.05
SrSO_2_ T1	80	81	87	81	73	86	< 0.05
SrSO_2_ T2	83	73	86	81^**b**^	75	88	< 0.05
CFTOE T0	0.18	0.14	0.22	0.18	0.17	0.20	NS
CFTOE T1	0.19	0.17	0.20	0.17	0.14	0.21	NS
CFTOE T2	0.17^**b**^	0.12	0.21	0.17^**a**^	0.16	0.19	NS
SFTOE T0	0.15	0.13	0.26	0.15	0.11	0.21	< 0.05
SFTOE T1	0.19^**c**^	0.14	0.36	0.13^**c**^	0.11	0.18	< 0.05
SFTOE T2	0.16	0.12	0.27	0.16	0.11	0.24	< 0.05
CSOR T0	1.00	0.98	1.05	0.96	0.93	1.00	< 0.05
CSOR T1	1.00^**a**^	0.99	1.17	0.96	0.95	0.99	< 0.05
CSOR T2	1.00^**a**^	0.99	1.07	1.00^**b**^	0.98	1.04	< 0.05

Values are expressed in median and interquartile ranges. a: *P* < 0.05 vs. T0; b: *P* < 0.05 T2 vs. T0-T1; c: *P* < 0.05 T1 vs. T0-T2, when compared in the same studied group

Abbreviations: cerebral regional oxygen saturation (CrSO_2_); splanchnic regional oxygen saturation (SrSO_2_); cerebral fractional tissue oxygenation extraction (CFTOE); splanchnic fractional tissue oxygenation extraction (SFTOE); cerebral-splanchnic oxygenation ratio (CSOR); not significant (NS)

In the splanchnic district BMSrSO_2_ pattern was flat and no differences (*P* > 0.05, for all) were found among pre-determined monitoring time-points. BM S FTOE at T1 was lower (*P* < 0.05, for both) than T2 and T0 whilst no differences (*P* > 0.05) were found between T0 and T2 (Table 3).

Cerebral-splanchnic hemodynamic pattern was characterized by a higher BM CSOR (*P* < 0.05, for both) at T2 and T1 than T0 whilst T2 and T1 did not differ (*P* > 0.05) (Table 3).

### NIRS monitoring after FM stimuli

In LP subjected to FM olfaction stimuli the FMCrSO_2_ pattern was characterized by a progressive increase with significantly higher (*P* < 0.05) values at T2 than T0. No differences (*P* > 0.05. for both) were found between T0-T1 and T1-T2, respectively.

FM C FTOE at T2 was significantly lower (*P* < 0.05) than T0, whilst no differences (*P* > 0.05. for both) were observed between T1-T2 and T0-T1, respectively (Table 3).

In the splanchnic district FMSrSO_2_ at T2 was lower than T0 and T1 (*P* < 0.05, for both), whilst no differences (*P* > 0.05) were found at T0-T1 time-points. FM S FTOE at T1 was lower (*P* < 0.05, for both) than T0 and T2, whilst no differences (*P* > 0.05) were found between T0 and T1 (Table 3).

Cerebral-splanchnic hemodynamic pattern was characterized by a higher FM CSOR (*P* < 0.05, for both) at T2 than T0 and T1, whilst T0 and T1 did not differ (*P* > 0.05) (Table 3).

### BM vs. FM NIRS parameters

In the cerebral district, BMCrSO_2_ was higher (*P* < 0.05, for both) at T0 and T2 than FMCrSO_2_, whilst no differences (*P* > 0.05) were observed at T1 (Table 3). Moreover, no differences (*P* > 0.05, for all) in C FTOE were observed between BM and FM groups at different monitoring time-points (Table 3).

In the splanchnic district, FMSrSO_2_ was higher (*P* < 0.05, for both) at T0 and T1 than BMSrSO_2_, whilst it was lower (*P* < 0.05) than BMSrSO_2_ at T2. FM S FTOE was lower (*P* < 0.05. for all) than BM S FTOE at all monitoring time-points (Table 3). Finally, BM CSOR was higher (*P* < 0.05, for all) than FM CSOR at all monitoring timepoints (Table 3).

## Discussion

Late preterm period is crucial for whole organ development, especially of the brain. At this time, the central nervous system (CNS) is at its maximum growing point in terms of brain weight, volume and synaptogenesis [30]. The fact is noteworthy, particularly for sense organ development such as visual and acoustic systems today screened by current standard-of-care procedures (i.e. acoustic oto-emissions and red pupillary reflex) [31–34]. Conversely, no progress has been achieved in the monitoring of olfactory function. In this respect, NIRS can constitute a promising noninvasive monitoring/diagnostic tool [9].

In the present study we showed that tissue oxygenation status and function as well as cerebral-splanchnic hemodynamic redistribution, in a cohort of LP infants, significantly changed before, during and after BM or FM olfaction stimuli. In detail, in LP subjected both to BM and FM olfaction stimuli: (i) CrSO_2_ increased at short-long term after olfaction stimuli (T1,T2), (ii) SrSO_2_ remained stable after BM olfaction, whilst it significantly decreased at short-long term after FM olfaction stimuli (T1,T2), (iii) CFTOE decreased at short term after BM/FM olfaction stimuli, whilst at long-term only after BM olfaction stimuli, (iv) SFTOE decreased after BM olfaction stimuli, whilst it increased after FM olfaction stimuli, and (v) a hemodynamic redistribution in favor of the brain at short-long term was shown. Finally, when NIRS parameters were compared according to different olfaction stimuli we found that FM olfaction stimuli correlated with lower CrSO_2_, with an increased splanchnic oxygenation status, tissue function and hemodynamic redistribution in favor of the gut.

Increased CrSO_2_ partially maps data in the literature: discrepancies related to different study-design and endpoints. On one hand, a beneficial effect on cerebral-splanchnic NIRS parameters has been reported in preterm infants fed by different regimens (BM, FM) and in different ways (bolus, gavage) [11, 16, 35]. On the other hand, changes in NIRS parameters have been shown after different olfaction stimuli response to various odors such as vanilla, distilled water, colostrum/maternal milk or formula milk in healthy full-term newborns [6, 9, 10, 12].

The finding of CrSO_2_ pattern after BM olfaction stimuli merits further consideration. In particular increased BMCrSO_2_: (i) offered additional support to olfaction CNS area development at the stage under investigation [6, 10], (ii) can be expression of frontal lobe activation associated with hemodynamic redistribution suggestive of neurovascular interaction, and (iii) corroborated the ability of LP infants to provide neurophysiological responses to the mother’s scent [6, 8, 10]. Altogether, it is reasonable to argue that among different milk regimens LP were able to distinguish BM scent, offering additional support to the unique BM properties including selective CNS area development such as olfaction. Additional advantages resided in an increased oxygen status and hemodynamic redistribution in favor of the brain, thus available for putative developmental processes. Further multicenter investigations in a wider population are therefore required.

In the present series we found, for the first time, that splanchnic oxygenation status, tissue activation and hemodynamic redistribution were higher in LP infants after FM olfaction stimuli. The NIRS pattern partially fits with previous observations showing differences between BM and FM regimens and under different administration modalities [15, 16].

The NIRS splanchnic pattern deserves further consideration. In particular increased SrSO_2_ and SFTOE are suggestive of a LP ability to distinguish a different olfaction stimulus from BM, one requiring a major oxygen request, tissue activation and subsequent hemodynamic response. The issue is noteworthy since similar NIRS patterns have been observed in infants fed by FM [14]. Therefore, it is tempting to argue that olfaction stimulus activates a cascade of behavioral and physiological events preparing the infants for feeding as well as their knowledge/memory of major energy consumption when fed by FM. These issues are further corroborated by a hemodynamic response suggestive of a redistribution in these cases in favor of the splanchnic district. Lastly, it is reasonable to infer that FM olfaction stimulus activates a response involving CNS and splanchnic oxygenation, tissue activation and hemodynamic at higher metabolic cost than BM. Further multicenter studies in a wider population aimed at elucidating the issue are required.

Finally, in the present study we identified a number of limitations such as: (i) the small number of cases studied recruiting LP alone instead of preterm and term ones, (ii) the stratification of LP infants for gender known to be different at the age of investigation, (iii) fixation problems of the sensors leading to movement artifacts and (iv) the reliability of NIRS measurements on the abdomen [36].

## Conclusions

In conclusion, the present findings providing early NIRS changes in cerebral and splanchnic districts after different BM/FM regimen odors, pave the way for further multicenter investigations using NIRS as a reliable tool for CNS and splanchnic development and response after olfaction stimuli.

## Electronic supplementary material

Below is the link to the electronic supplementary material.

Supplementary Material 1

## Data Availability

The data that support the findings of this study are available from the corresponding author, [DG], upon request.

## Abbreviations

BM: Breast milk
NICU: Neonatal intensive care unit
NIRS: Near infrared spectroscopy
rSO_2_: Regional oxygen saturation
FTOE: Fraction of tissue oxygen extraction
FM: Formula milk
IUGR: Intrauterine growth retardation
LP: Late preterm
GA: Gestational age
BW: Birthweight
C: Cerebral
S: Splanchnic
SaO_2_: Arterial oxygen saturation
CSOR: Cerebral/splanchnic oxygenation ratio
RBC: Red blood cell count
Hb: Hemoglobin blood concentrations
Ht: Hematocrit rate
pCO_2_: Partial carbon dioxide venous pressure
pO_2_: Partial oxygen venous pressure
BE: Base excess
PROM: Premature rupture of membrane
GDM: Gestational diabetes mellitus
RDS: Respiratory distress syndrome
MV: Mechanical ventilation
NIV: Non-invasive ventilation
BPD: Bronchopulmonary dysplasia
PDA: Persistent patent ductus arteriosus
IVH: Intraventricular hemorrhage
PVL: Periventricular leukomalacia
EOS: Early onset sepsis
LOS: Late onset sepsis
ROP: Retinopathy of prematurity
CNS: Central nervous system
