# Supplementary material for: Short-long term near infrared spectroscopy patterns after different milk regimens olfactory stimuli in late preterms
Source: Ital J Pediatr. 2025 Apr 12;51:113. doi: 10.1186/s13052-025-01912-0 (PMC11994012; doi:10.1186/s13052-025-01912-0)
Supplement: Supplementary file 1 — Supplementary Material 1 [file 13052_2025_1912_MOESM1_ESM.docx]

Chieti, February 6^th^, 2025

To:

Prof. Giovanni Corsello

Editor in Chief

Italian Journal of Pediatrics

Dr. Gregorio Serra

Associate Editor

Italian Journal of Pediatrics

**Object: MS# ITJP-D-24-00749**

Dear Editor,

enclosed please find the revised version of the MS entitled *“*Short-Long Term Near Infrared Spectroscopy Patterns after different Milk Regimens Olfactory Stimuli in Late Preterms*”* by Di Battista C. et al.

We want to thank you and the reviewers for kind comments and useful suggestions that have been taken into the due account in the present MS revised version.

Changes are marked in yellow. In particular:

***Reviewer #1:***

- *This study is more oriented on the evaluation of the presence of a brain activation response to stimulus than on functional cerebral maturation, thus in Discussion references to the concepts of functional development including comparison with screening tests for non sensineural auditory pathology and normal eye structure (not visual function or maturation), should be reformulated.*

According to the reviewer’s suggestion reference list has been updated as requested. We trust that now is clearer.

- *I suggest to merge 2.5 and 2.6 sections titled cerebral assessment and neurological examination.*

According to the reviewer’s suggestion 2.5 and 2.6 sections have been merged. We trust that now is clearer.

**Reviewer #2:**

- *Congratulations to the authors! The study is well conducted, with a robust statistical analysis and evaluation of the results. The paper is also well presented. Only the results section seems excessively full of acronyms, which hamper the flowing and immediacy in the understanding.*

We want to thank the reviewer for kind comments and useful suggestions. However, we were able to skip from all MS sections redundant acronyms maintaining only those needed. We trust that now is clearer.

- *Apart from the suggestion of improving such formal aspect, I only further suggest a few and minor changes:*
  - *Abstract: Add the acronym NIRS after the first time it appears in the background*
  - *Keywords: change preterm with premature to differentiate from the title*
  - *Methods - NIRS monitoring*
    - *SrSO2, explain in full*
    - *rSO2, which included in the formula to obtain fractional tissue oxygen extraction, must be explained*
  - *Results*
    - *Population: Add a comma after "26.3%"*
    - *Neonatal outcomes: write in uppercase "Caesarean section"*
    - *the need for surfactant administration (add "for")*
    - *Please clarify the title of the paragraph "NIRS monitoring after BM" (add stimuli, if the authors are referring to this, for better clarity)*
    - *BMCFTOE, explain in full*
  - *Discussion*
    - *MF, please amend*
    - *CNS, specify in full*

According to the reviewer’s suggestion, MS has been changed in all sections as requested. We trust now it is clearer.

- *Finally, a figure which may attract the attention of the reader, explaining in a synthetic and more impactful way the best nirs profiles highlighted in newborns after stimulation with breast milk, could be useful to enrich the work and make it more captivating.*

We want to thank the reviewer for useful suggestion. However, in order to avoid redundant data we trust that Table 3 is able to offer a general and detailed description of the data obtained. In any case, we are available to offer an additional figure if needed.

**Editorial Board comments:**

- *Please provide City and country in the affiliation for author(s) (Aff7) in the manuscript.*

We included affiliation details in the title page. In this regard, in the R1 version submission process the system doesn’t allow us or we are not able to access to authors page in order to verify their affiliation. We apologize for this.

- *Authors' contributions*

We included Author’s contribution at the end of Discussion section and differentiated the duplicated initials of the authors. We trust that now is clearer.

- *Missing Figures*

Figure 1 has been uploaded in the revised version of the MS. We trust that now is clearer.

We trust that now that the MS in its present form will meet the requirements for publication on Your Journal.

Best regards

Diego Gazzolo MD, PhD
